# Novel sporadic c.2687G>T (p.Gly896Val) CYLD mutation in multiple trichoepitheliomas^؆^

**DOI:** 10.1016/j.abd.2025.501282

**Published:** 2026-01-15

**Authors:** Ahmet Uğur Atılan, Niyazi Cetin

**Affiliations:** Department of Dermatology, School of Medicine, Pamukkale University, Denizli, Turkey

Dear Editor,

Multiple trichoepithelioma (MTE) is a rare autosomal dominant neoplasm characterized by multiple skin-colored papules, primarily on the face.^1^ It is associated with mutations in the CYLD tumor suppressor gene and originates from the pilosebaceous unit.^2^ Variant mutations in the CYLD gene are associated with diseases in the CYLD cutaneous spectrum, such as Brooke-Spiegler syndrome, familial cylindromatosis, and multiple familial trichoepithelioma.^3^ While familial cases predominate, de novo CYLD mutations are rarely documented, limiting genotype-phenotype correlations and genetic counselling guidance. Here, we report a case of multiple trichoepithelioma presenting with a new sporadic missense mutation in the CYLD gene.

A 31-year-old woman presented with asymptomatic papular lesions on the nasolabial folds, nose, and forehead, which had first appeared during the first month of life and progressively increased in number over time (Fig. 1). Lesions began perinasally and later involved the scalp and extremities. No additional skin tumor, such as cylindroma or spiradenoma, was detected. The patient denied a family history of similar lesions, and there was no known consanguinity. Physical examination revealed multiple, firm, asymptomatic, skin-colored papular lesions of 0.2‒0.4 cm in diameter on bilateral nasal margins and forehead (Fig. 1). Multiple soft nodular lesions were observed on the occipital region and scalp (Fig. 2), along with a 1.4 cm nodule on the extensor surface of the right elbow and hyperkeratotic papules on the dorsum of the left toes. Dermatoscopic examination revealed a regular architecture with regular borders, thin, irregular vessels located peripherally, and opaque white areas on the lesion, which were suggestive of trichoepithelioma (Fig. 3). No dermoscopic signs of basal cell carcinoma (BCC) or sebaceous adenoma were present. Laboratory investigations showed no abnormalities. A punch biopsy was obtained from a lesion on the nasal margin with clinical differential diagnoses including adenoma sebaceum, syringoma, and trichoepithelioma. Histopathological examination disclosed features consistent with trichoepithelioma, characterized by basaloid cell islands, papillary mesenchymal structures, and cysts containing lamellar keratin within the dermis. DNA sequence analysis covering all coding exons (9–20) and exon-intron boundaries of the CYLD gene was performed using bidirectional capillary Sanger sequencing, as previously described.^2^, ^3^ This analysis revealed a heterozygous variant, NM_015247.2:c.2687 G > G>T (p.Gly896Val), located in exon 20, with other previously reported mutations also shown in Fig. 4.^4^, ^5^ The patient was referred for genetic counselling. Due to financial constraints, laser treatment was not performed. Instead, selected lesions were treated with cryotherapy and electrocautery; however, these interventions were discontinued due to unsatisfactory cosmetic results. The patient continues to be under regular clinical follow-up.

**Fig. 1:**
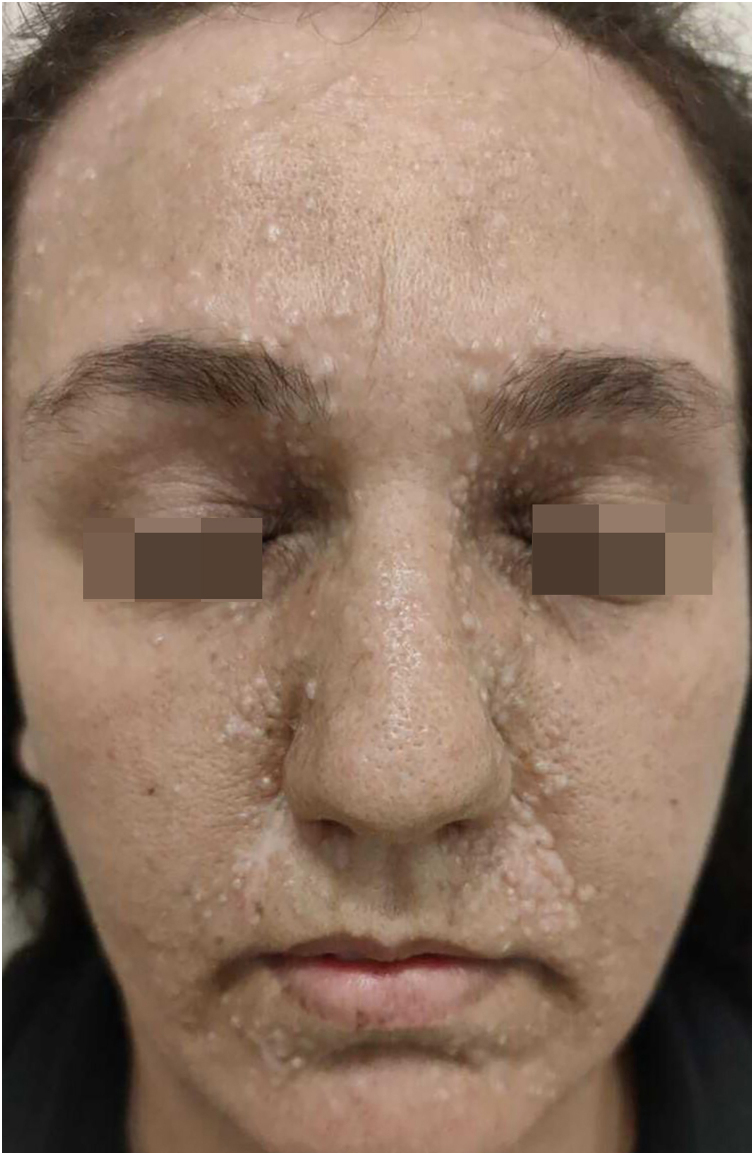
Multiple skin-colored papules (0.5–2 mm) symmetrically distributed over the central face, consistent with trichoepitheliomas.

**Fig. 2:**
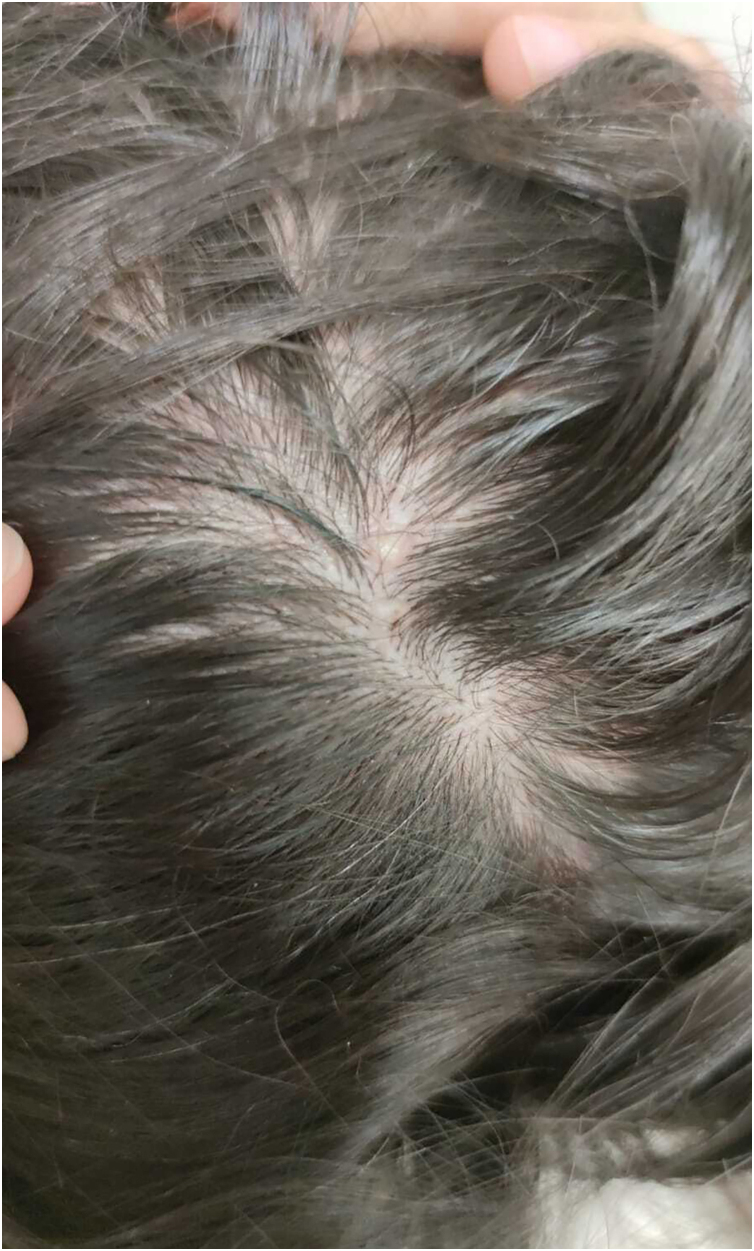
Multiple soft nodules on the occipital region, clinically consistent with multiple trichoepitheliomas.

**Fig. 3:**
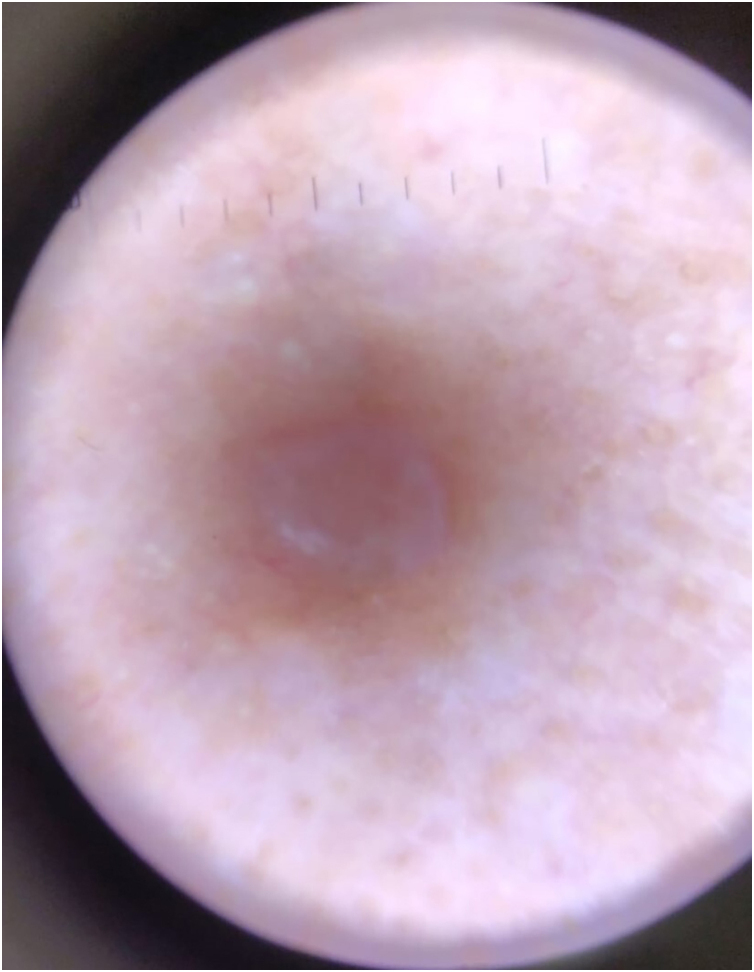
Dermoscopy demonstrating regular lesion architecture with well-defined borders, peripheral thin irregular vessels, and central opaque white areas.

**Fig. 4:**
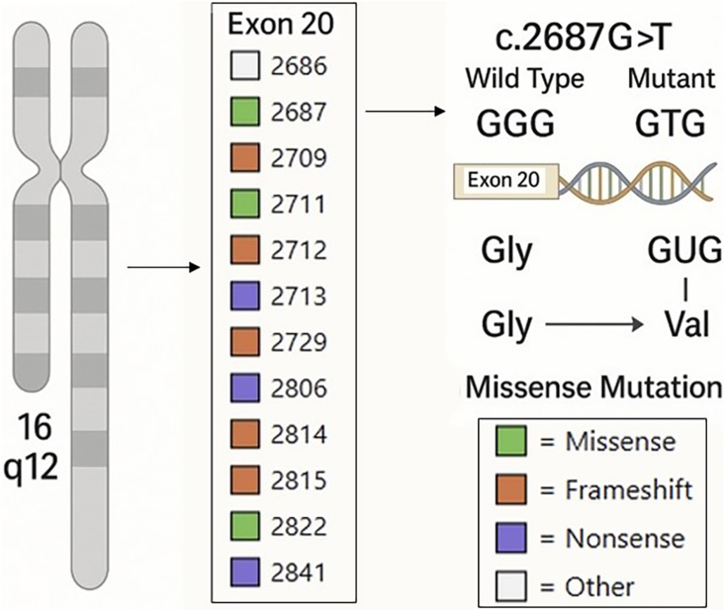
Schematic of CYLD exon 20 showing the novel NM_015247.2:c.2687 G > G>T (p.Gly896Val) missense mutation identified in our patient (GGG → GTG; Gly → Val). Previously reported MTE-associated mutations at this exon are also displayed.^4^, ^6^ Mutation types are color-coded: Green, Missense; Orange, Frameshift; Purple, Nonsense; Grey, Other (Large deletions and rearrangements). Gly, Glycine, Val, Valine, MTE, Multiple Trichoepithelioma.

CYLD encodes a deubiquitinase regulating NF-κB and other pathways; loss-of-function mutations underlie several adnexal tumor syndromes.^4^, ^6^ Mutations in the CYLD gene usually occur between exons 9 and 20 and have different phenotypic consequences.^5^ Various CYLD gene mutations, such as splicing, frameshift, missense, and nonsense, which have been identified in recent years, have contributed significantly to the understanding of the phenotypic diversity of diseases such as Multiple Trichoepithelioma and Brooke-Spiegler syndrome in the CYLD-associated cutaneous syndrome spectrum.^2^, ^3^ Generally, missense mutations of the CYLD gene have been linked to less phenotypic variation compared to other mutation types, and the majority of these are multiple familial trichoepithelioma cases.^5^ Although several mutations have been reported in exon 20 of the CYLD gene located at 16q12–q13, only a single substitution at nucleotide position c.2687 ‒ namely c.2687 G > G>C (p.Gly896Ala) ‒ has previously been described by España et al., in a familial case of multiple trichoepithelioma from Spain.^7^ CYLD gene mutations, which have a well-defined autosomal dominant inheritance pattern, can also be seen sporadically, albeit rarely.^8^, ^9^

In conclusion, to our knowledge, this is the first sporadic report of the NM_015247.2:c.2687 G > G>T (p.Gly896Val) variant; prior familial reports were limited to a Spanish pedigree. Although parental testing could not be performed and de-novo status therefore remains unconfirmed, this finding extends the known mutational spectrum of CYLD-associated cutaneous tumours and underlines the potential value of CYLD genetic testing even in non-familial cases. Reporting such cases not only contributes to the development of genetic counselling but also improves our understanding of the molecular pathogenesis of CYLD-associated skin diseases. Further molecular research is required to clarify the functional impact of novel CYLD variants in skin tumourigenesis and to confirm the pathogenicity of the p.Gly896Val substitution.

## ORCID ID

Niyazi Cetin: 0009-0009-9280-6135

## Authors' contributions

Ahmet Uğur Atılan: Study design and conception; critical literature review; final approval of the final version of the manuscript.

Niyazi Cetin: Writing of the manuscript or critical review of important intellectual content; critical literature review.

## Financial support

There was no financial support.

## Research data availability

Does not apply.

## Conflicts of interest

None declared.
